# Social Media Use and Psychological Symptoms Among Older Adults: Sequential Indirect Associations Through Attitudes Toward Aging and Basic Psychological Need Satisfaction

**DOI:** 10.3390/bs16101765

**Published:** 2026-09-28

**Authors:** Liqing Liu, Tong Yang, Jiale Wu, Jiaru Qin, Ziyu Wang

**Affiliations:** 1 Faculty of Psychology, Tianjin Normal University, Tianjin 300387, Chinawujiale@stu.tjnu.edu.cn (J.W.); qinnjiaru@163.com (J.Q.); zy19163759014@163.com (Z.W.); 2 Key Research Base of Humanities and Social Sciences of the Ministry of Education, Academy of Psychology and Behavior, Tianjin Normal University, Tianjin 300387, China; 3 Tianjin Social Science Laboratory of Students’ Mental Development and Learning, Tianjin 300387, China; 4 Key Laboratory of Modem Teaching Technology, Ministry of Education, Shaanxi Normal University, Xi’an 710062, China

**Keywords:** older adults, social media use, attitudes toward aging, basic psychological needs, psychological symptoms

## Abstract

With rapid population aging, older adults’ psychological symptoms are a growing public concern. This cross-sectional study examined associations among social media use, attitudes toward aging, basic psychological need satisfaction, and psychological symptoms in 325 older adults recruited by convenience sampling in Tianjin, China. Eligibility was restricted to older adults who used at least one social media platform. Participants completed self-report measures of the study variables. Social media use was positively associated with attitudes toward aging and basic psychological need satisfaction and negatively associated with psychological symptoms. In a cross-sectional indirect-effects model, the direct association between social media use and psychological symptoms was not significant after attitudes toward aging and basic psychological need satisfaction were included. The indirect association through basic psychological need satisfaction was statistically significant, whereas the indirect association through attitudes toward aging alone was not. The sequential indirect association involving attitudes toward aging and basic psychological need satisfaction was consistent with the hypothesized model, but the cross-sectional design precludes conclusions about temporal ordering or causality. These findings suggest potential psychosocial correlates linking social media use with psychological symptoms among older social media users and highlight directions for future longitudinal research on healthy aging in digital contexts.

## 1. Introduction

### 1.1. Psychological Symptoms in Older Adults

Population aging constitutes one of the most significant demographic transformations of the 21st century, placing the promotion of active and healthy aging at the forefront of public health agendas. Contemporary perspectives increasingly recognize that older adulthood is not merely a period of physiological decline but also a stage characterized by ongoing personal growth, social engagement, and adaptive capacity (Ayoubi-Mahani et al., 2023). Indeed, older adults are progressively investing their time and energy in self-care, community participation, and the maintenance of meaningful social ties, both offline and online.

However, the transition into later life frequently coincides with major psychosocial stressors, including bereavement, retirement, and geographic separation from family networks. If unaddressed, these transitions can precipitate a contraction of social networks and elevate the risk of loneliness, anxiety, and depressive symptoms (Cornwell & Waite, 2009; Shao et al., 2022). Rather than being inherent deficits of aging, these psychological difficulties represent critical challenges that may be mitigated through accessible social resources.

The rapid proliferation of digital and age-friendly technologies has been accompanied by older adults’ adoption of novel strategies for social participation. Social media platforms, in particular, offer flexible avenues for maintaining pre-existing relationships, accessing informational support, and engaging in community life, thereby counterbalancing the constraints of physical mobility (Chen et al., 2022; Y. Liu et al., 2026). Consequently, social media has become an important channel through which older adults proactively manage their social well-being. Despite its growing prevalence, however, the psychological mechanisms that may be involved in the association between social media use and psychological symptoms—whether beneficial or detrimental—remain insufficiently elucidated. Addressing this gap may inform the development of targeted interventions involving digital tools for psychological symptoms in later life.

### 1.2. Social Media and Psychological Symptoms

Social media has substantially expanded opportunities for online social participation among older adults. Compared with traditional face-to-face interaction, online communication can compensate for age-related declines in physical functioning and mobility by enabling individuals to maintain existing social ties and develop new social connections (Dehi Aroogh & Mohammadi Shahboulaghi, 2020).

Several studies have found that older adults could benefit from social media use. Research has shown that social media use by older adults can enhance communication with friends, family, and others, helping to reduce feelings of loneliness, increase self-efficacy, and attenuate psychological symptoms (Fokkema & Knipscheer, 2007; H. Wang et al., 2022). Findings from another study of older Korean adults suggest that increasing older adults’ use of digital media may relate to positive attitudes toward aging and healthy aging (Pan et al., 2024). These findings imply that social media may function as a valuable resource for psychological adaptation in later life.

However, the relationship between social media use and psychological symptoms is not uniformly favorable. Studies conducted among broader and often younger populations have shown that social media use may expose individuals to social comparison, misinformation, negative online interactions, and excessive information overload, all of which can undermine psychological well-being (Keles et al., 2020; O’Day & Heimberg, 2021). Whether these adverse processes operate similarly among older adults remains less clear. Although some research suggests that older adults may use social media primarily to maintain existing social relationships rather than for entertainment or self-presentation (Chen et al., 2022), the present study did not assess usage motives, content type, passive use, or problematic use. Moreover, because only existing social media users were eligible, the current findings cannot determine whether using social media itself is associated with better psychological outcomes. The overall association between social media use and psychological symptoms therefore remains inconclusive, and the indirect pathways observed in this study should be interpreted as conditional and specific to the present sample of older adult social media users.

### 1.3. Attitudes Toward Aging and Basic Psychological Needs Satisfaction as Sequential Mediators

To understand the potential indirect associations among social media use, attitudes toward aging, basic psychological needs satisfaction, and psychological symptoms in older adults, this study proposes a chain mediation model. Specifically, we examined whether social media use was associated with older adults’ cognitive appraisals of their own aging; whether these appraisals were associated with the satisfaction of basic psychological needs; and whether greater need satisfaction was associated with attenuated psychological symptoms. This sequential set of associations offers the potential for greater explanatory power than single-mediation models and appears more closely aligned with the psychological development characteristics of older adults.

#### 1.3.1. Attitudes Toward Aging as a Cognitive Pathway

Attitudes toward aging refer to individuals’ cognitive evaluations and subjective interpretations of their own aging process, encompassing perceptions of psychosocial loss, physical change, and psychological growth (Laidlaw et al., 2007). These attitudes are linked to the psychological symptoms in later life: older adults with more positive aging perceptions tend to report lower levels of depression and anxiety, whereas negative attitudes are consistently associated with heightened psychological distress (Bryant et al., 2012; Seow et al., 2025; D. W. Lai, 2009; X. G. Wang et al., 2021). Stereotype Embodiment Theory (Levy, 2009) provides a framework for understanding how such attitudes are formed and internalized. Age-related beliefs acquired from the social environment become incorporated into self-perceptions over time, subsequently influencing behaviors and psychological outcomes. Previous studies showed that greater participation in online social activities has been linked to more favorable self-perceptions of aging (Mei et al., 2024; Sabatini et al., 2023). Therefore, social media use may be associated with lower psychological symptoms partly because it is related to more positive attitudes toward aging.

#### 1.3.2. Basic Psychological Needs Satisfaction as a Motivational Pathway

Self-determination theory (Ryan & Deci, 2000) posits that autonomy, competence, and relatedness are fundamental psychological needs whose satisfaction is essential for well-being. Multiple studies have demonstrated that fulfilling these basic psychological needs significantly enhances the quality of life and alleviates psychological symptoms among older adults. On the other hand, when these needs are thwarted, the risk of depression increases significantly, leading to a reduction in overall well-being (Behzadnia et al., 2020; Cantarero et al., 2020). For older adults, social media use may serve as an effective means of satisfying these needs. Unlike younger individuals, older adults are less likely to become addicted; instead, it could become a positive way for them to maintain social contacts and establish new social connections (Zhao & Liu, 2020). Maintaining contact with others and consolidating social networks can enhance autonomy and sense of belonging. Moreover, learning how to use social media and acquiring new skills can increase older adults’ sense of competence. Therefore, social media use may be linked to reduced psychological symptoms by satisfying basic psychological needs.

#### 1.3.3. The Chain Mediation Model

Although attitudes toward aging and basic psychological need satisfaction have each been examined as independent mediators, a sequential model better captures the cognitive and motivational processes involved. According to self-determination theory, the fulfillment of basic psychological needs is not automatic; it is shaped by how individuals interpret and evaluate their social environment (Ryan & Deci, 2000). In later life, attitudes toward aging function as a critical cognitive lens through which older adults appraise whether their social interactions and daily activities can genuinely satisfy their needs for autonomy, competence, and relatedness. Empirical evidence supports this sequential pathway. Nam and Cho (2018) found that positive aging attitudes are closely associated with higher levels of basic psychological need satisfaction, which in turn predict successful aging outcomes. Qualitative evidence further indicates that negative age-related stereotypes directly compromise older adults’ perceived competence and autonomy, reducing intrinsic motivation and behavioral engagement (Palombi et al., 2025). Additionally, Kuo and Chang (2024) demonstrated that aging self-perceptions moderate the relationship between basic psychological need satisfaction and well-being, suggesting that attitudes toward aging shape the psychological benefits individuals can derive from need-fulfilling experiences.

The findings suggest that social media use may be linked to more positive attitudes toward aging. These positive attitudes, in turn, appear to be associated with greater satisfaction of basic psychological needs. Moreover, higher satisfaction of these needs is correlated with fewer psychological symptoms. Unlike single-mediator models, this sequential framework, linking cognition to motivation, offers a more comprehensive understanding of the relationships among social media engagement, attitudes toward aging, need satisfaction, and psychological symptoms in older adults. However, due to the cross-sectional nature of the data, the proposed sequence of variables should be viewed as a theoretical model rather than definitive evidence of causal relationships.

### 1.4. The Present Study and Hypothesis

Despite growing interest in the relationship between social media use and mental health among older adults, three important gaps remain. First, empirical findings regarding the association between social media use and psychological symptoms remain inconsistent. Second, limited attention has been paid to the psychological mechanisms underlying this relationship. Third, existing studies rarely integrate cognitive and motivational processes within a unified theoretical framework. To address these gaps, the present study draws upon Stereotype Embodiment Theory and Self-Determination Theory to test a theory-driven sequential indirect association among older adults. The key contribution is not the inclusion of two mediators per se, but the specification and testing of a sequential model in which attitudes toward aging precede basic psychological need satisfaction, thereby integrating a cognitive representation of aging with a motivational process. Importantly, this study does not compare users with non-users; rather, it examines whether the frequency of social and information-related social media behaviors among existing older social media users is associated with attitudes toward aging, basic psychological need satisfaction, and psychological symptoms.

Accordingly, the following hypotheses are proposed:

**H1.**  *Social media use is negatively associated with psychological symptoms among older adults who use social media.*

**H2.**  *The association between social media use and psychological symptoms is statistically accounted for, in part, by an indirect pathway through attitudes toward aging.*

**H3.**  *The association between social media use and psychological symptoms is statistically accounted for, in part, by an indirect pathway through basic psychological need satisfaction.*

**H4.**  *Social media use is indirectly associated with psychological symptoms through a sequential statistical pathway involving attitudes toward aging and basic psychological need satisfaction.*

## 2. Materials and Methods

### 2.1. Participants

This study employed a convenience sampling method to recruit older adults from senior activity centers located in relatively large communities and public parks across four urban and suburban districts in Tianjin, China. The districts and sites were selected based on accessibility and the willingness of local administrators to cooperate. Research assistants approached older adults individually to explain the study, and only those who reported using at least one social media platform, such as WeChat or Douyin, were eligible to participate. The survey was administered through a combination of online and offline channels. For the offline survey, written informed consent was obtained, and participants were explicitly informed that they could withdraw at any time without penalty. Offline questionnaires were completed in a quiet setting, either self-administered or read aloud by the researcher. For the online survey, participants were informed that submission of the questionnaire would be considered as providing consent to participate on Wenjuanxing (Changsha Ranxing Information Technology Co., Ltd., Changsha, China). A total of 372 participants completed the survey. Of these, 20 were younger than 60 years, and 35 exhibited straight-line response patterns, defined as selecting the same option for 25 or more consecutive items. Eight participants met both exclusion criteria. After excluding these 47 unique cases, the final analytical sample consisted of 325 participants (116 males, 209 females; age range 60–88 years). Gender was coded as 1 = male, 2 = female. Educational level was coded as 1 = illiteracy, 2 = primary school, 3 = junior high school, 4 = high school, 5 = bachelor’s degree, and 6 = master’s degree and above. The study was approved by the Ethics Committee of Tianjin Normal University (Approval No. 2025123112) and conducted in accordance with the Declaration of Helsinki.

### 2.2. Measurements

#### 2.2.1. Basic Psychological Needs

Basic psychological needs in older adults were assessed using the Basic Psychological Needs Scale (BPNS) developed by Gagné (2003). The Chinese version of the BPNS was translated and revised by J. S. Liu et al. (2013). To account for cultural differences, two items from the original scale were removed: “I feel like I am free to decide for myself how to live my life” and “I consider the people I regularly interact with to be my friends” (J. S. Liu et al., 2013). The revised scale consists of 19 items across three dimensions (autonomy, competence, and relatedness), each rated on a 7-point scale ranging from 1 (not at all) to 7 (very much), with 9 items being reverse-scored. Higher scores indicate greater satisfaction of basic psychological needs, and lower scores indicate lower satisfaction. The Cronbach’s alpha for the scale in this study was 0.786. For the three dimensions, composite reliability values were 0.662 (autonomy), 0.579 (competence), and 0.733 (relatedness), and average variance extracted (AVE) values were 0.283, 0.315, and 0.319, respectively. The relatively low AVE values for the BPNS subscales suggest limited convergent validity in this sample. Future studies should further examine the psychometric properties of the BPNS among Chinese older adults. Exploratory factor analysis (EFA) using principal axis factoring with varimax rotation was specified a priori for a three-factor solution based on the theoretical structure; the three factors together explained 46.88% of the total variance. Factor loadings ranged from 0.443 to 0.690 for Factor 1, −0.571 to 0.700 for Factor 2, and 0.489 to 0.551 for Factor 3. Seventeen of the 19 items had salient loadings (≥0.40) on their intended factors. However, the third factor contained only two salient items, and the subscale reliabilities and AVE values were relatively low. Therefore, the hypothesized three-factor structure was only partially supported and should be interpreted with caution. The Kaiser–Meyer–Olkin (KMO) value was 0.806, and Bartlett’s test of sphericity was significant, χ^2^ = 1425.917, *p* < 0.001.

#### 2.2.2. Psychological Symptoms

Given that the widely used Symptom Checklist-90 (SCL-90) is quite lengthy, which may cause fatigue or even lead to dropout among older adult participants, this study adopted its abbreviated version, the SCL-10, to assess psychological symptoms. The SCL-10, which is a short form of the SCL-90 developed by Derogatis et al. (1973), has been shown to perform almost as well as the full version (Rosen et al., 2000; Strand et al., 2003). It consists of 10 items, each rated on a 5-point scale. Higher total scores indicate more severe psychological symptoms and, accordingly, poorer mental well-being. To our knowledge, no Chinese version of the SCL-10 has been formally validated specifically in Chinese older adults. Therefore, the Chinese SCL-10 used in this study was adapted from the validated Chinese version of the SCL-90 by selecting the 10 items corresponding to the SCL-10 as defined by Strand et al. (2003). In the present sample, internal consistency was acceptable (Cronbach’s alpha = 0.874; composite reliability = 0.853), but the AVE was 0.374, indicating limited convergent validity. EFA using principal axis factoring extracted one factor, accounting for 30.87% of the total variance, with all 10 items loading on this factor (loadings = 0.301–0.753). These results provide preliminary but limited support for the unidimensional structure of the Chinese SCL-10. The KMO value was 0.802, and Bartlett’s test of sphericity was significant, χ^2^ = 1004.619, *p* < 0.001.

#### 2.2.3. Social Media Use

Older adults’ social media use was assessed using the Social Media Use Behavior Scale for Older Adults developed by Deng (2021). Prior research with Chinese older adults has supported the scale’s construct validity and two-factor structure. The scale consists of two subscales: Social Behavior (15 items) and Information Behavior (10 items). Both subscales were included in the present study to comprehensively capture older adults’ social media engagement. Items were rated on a 5-point scale ranging from 1 (never) to 5 (always), with higher scores indicating more frequent social media use. Cronbach’s alpha was 0.950 for the total scale, 0.933 for the Social Behavior subscale, and 0.904 for the Information Behavior subscale.The composite reliability values were 0.941 for the Social Behavior subscale and 0.916 for the Information Behavior subscale, with average variance extracted values of 0.517 and 0.523, respectively, supporting convergent validity. The two subscales were combined into a total score for the primary analysis, as both dimensions reflect different facets of the broader construct of social media use behavior, and the present study focused on the overall pattern of social media engagement rather than distinguishing between specific types of use. EFA using principal axis factoring with varimax rotation yielded two factors based on the theoretical structure, explaining 53.62% of the total variance. Factor loadings ranged from 0.440 to 0.791 for Factor 1 and 0.487 to 0.845 for Factor 2 (salient loadings ≥ 0.40). All 25 items had a salient loading on at least one factor, although several items showed cross-loadings. These results provide preliminary support for a two-factor solution, but the correspondence between the extracted factors and the originally proposed dimensions was only partial. The KMO value was 0.919, and Bartlett’s test of sphericity was significant, χ^2^ = 6857.995, *p* < 0.001.

#### 2.2.4. Attitudes Toward Aging

The revised Attitudes Toward Aging Questionnaire was used to assess older adults’ attitudes toward aging. The original scale was developed by Laidlaw et al. (2007) and later translated and revised by Y. F. Huang et al. (2010). The Chinese version of the AAQ has demonstrated acceptable reliability and validity for the total score (Che et al., 2024; Y. F. Huang et al., 2010). The scale consists of 24 items across three dimensions: psychosocial loss, physical change, and psychological growth. Items are rated on a 5-point Likert scale (1 = strongly disagree, 5 = strongly agree). Higher scores in the psychosocial loss subscale indicate a more negative change in the psychosocial aspect of aging. Therefore, the scores in this subscale were reverse-coded when calculating the total score. In contrast, higher scores in the psychological growth and physical change subscales reflect a more positive change. Consequently, higher total scores indicate more positive attitudes toward aging. In the present study, Cronbach’s alpha for the scale was 0.815. Cronbach’s alpha values for the psychosocial loss, physical change, and psychological growth dimensions were 0.750, 0.853, and 0.810, respectively. Composite reliability values were 0.820, 0.874, and 0.868, and AVE values were 0.366, 0.501, and 0.487. EFA using principal axis factoring with varimax rotation was specified a priori for a three-factor solution based on the theoretical structure, explaining 46.62% of the total variance. Factor loadings ranged from 0.400 to 0.759 for Factor 1, 0.594 to 0.693 for Factor 2, and 0.466 to 0.596 for Factor 3. Twenty-two of the 24 items had salient loadings (≥0.40) on their intended factors, providing preliminary support for the three-factor structure. The KMO value was 0.874, and Bartlett’s test of sphericity was significant, χ^2^ = 2784.723, *p* < 0.001.

### 2.3. Statistical Analysis

SPSS 26.0 (IBM Corporation, Armonk, NY, USA) was used to organize and analyze the data. To test the hypothesized mediation model, Model 6 of the PROCESS macro for SPSS, version 3.5 (Andrew F. Hayes, University of Calgary, Calgary, AB, Canada), was employed, which is suitable for examining chain mediation effects. Mediation analysis was conducted with 5000 bootstrap resamples, and bias-corrected 95% confidence intervals were computed. Assumptions for mediation (linearity, homoscedasticity, and the absence of multicollinearity) were checked and met. All continuous variables were standardized prior to analysis. In this model, social media use was designated as the independent variable, while the level of psychological symptoms served as the dependent variable; attitudes toward aging and basic psychological needs satisfaction were entered as mediating variables. Additionally, age, gender, and educational level were included as control variables to account for potential confounding effects according to prior findings (Guzman et al., 2025; J. Huang et al., 2025), consistent with contemporary guidance on confounder selection (Sun et al., 1996; VanderWeele, 2019).

## 3. Results

### 3.1. Common Method Bias Tests

This study collected the data using a questionnaire survey method, and exploratory factor analysis was used to test for potential common method bias (Zhou & Long, 2004). Specifically, the Harman single-factor test was performed on the data. The unrotated exploratory factor analysis revealed 19 factors with eigenvalues greater than 1. Notably, the first factor accounted for 20.26% of the variance, which is below the 40% threshold. However, because all measures were self-reported at a single time point, this test alone cannot rule out common method bias. Therefore, although these results are not indicative of severe common method bias, common method bias cannot be definitively excluded.

### 3.2. Descriptive Statistics and Correlation Analysis

Correlation analysis was conducted on social media use, attitudes toward aging, basic psychological needs satisfaction, and psychological symptoms in older adults, using SPSS 26.0.

As shown in Table 1, social media use was significantly and positively correlated with both attitudes toward aging (*r* = 0.316, *p* < 0.001) and basic psychological need satisfaction (*r* = 0.423, *p* < 0.001), while showing a significant negative correlation with psychological symptoms (*r* = −0.201, *p* < 0.001). Furthermore, attitudes toward aging and basic psychological need satisfaction were significantly and positively intercorrelated (*r* = 0.392, *p* < 0.001). Both variables were also found to be significantly and negatively associated with psychological symptoms (*r* = −0.207 and *r* = −0.427, respectively).

### 3.3. Mediation Analysis

The regression analysis was conducted with gender, age, and education level included as control variables. The results (see Table 2) indicate that social media use significantly and positively predicts attitudes toward aging (β = 0.312, *SE* = 0.053, *p* < 0.001) as well as basic psychological needs satisfaction (β = 0.317, *SE* = 0.056, *p* < 0.001). Additionally, attitudes toward aging among older adults positively predict basic psychological needs satisfaction (β = 0.324, *SE* = 0.056, *p* < 0.001). Furthermore, basic psychological needs satisfaction significantly and negatively predicts psychological symptoms (β = −0.334, *SE* = 0.048, *p* < 0.001). In contrast, social media use was not a significant direct predictor of psychological symptoms (β = −0.004, *SE* = 0.051, *p* = 0.946).

The indirect effect of attitudes toward aging and basic psychological needs satisfaction on the relationship between social media use and psychological symptoms was examined using the SPSS Macro PROCESS Model 6. In this study, social media use was the independent variable, psychological symptoms served as the dependent variable, and both attitudes toward aging and basic psychological needs satisfaction were treated as mediating variables. The results are shown in Table 3.

The total effect of social media use on psychological symptoms was found to be significant (total effect = −0.157, *SE* = 0.049, 95% *CI* [−0.254, −0.059]). Additionally, the indirect effect of basic psychological needs satisfaction on the relationship between social media use and psychological symptoms was also significant (indirect effect = −0.106, *SE* = 0.026, 95% *CI* [−0.161, −0.060]). Furthermore, the chain mediation of attitudes toward aging and basic psychological needs satisfaction in the association between social media use and psychological symptoms was significant (indirect effect = −0.034, *SE* = 0.011, 95% *CI* [−0.057, −0.017]). In contrast, the indirect effect of the attitudes toward aging on the relationship between social media use and psychological symptoms was not significant (indirect effect = −0.013, *SE* = 0.016, 95% *CI* [−0.047, 0.017]). Therefore, H2 was not supported, as the confidence interval for the indirect effect through attitudes toward aging alone included zero. In summary, the association between social media use and psychological symptoms showed a significant indirect association through basic psychological needs satisfaction. In addition, the sequential indirect association through attitudes toward aging and basic psychological needs satisfaction was also significant (see Table 3 and Figure 1). Given the cross-sectional design, these patterns should not be interpreted as evidence of causal mediation.

### 3.4. Alternative Reverse-Order Mediation Model

Given that the cross-sectional design precludes establishing the temporal ordering of attitudes toward aging and basic psychological needs satisfaction, we conducted an additional analysis in which the order of the two mediators was reversed. Specifically, basic psychological needs satisfaction was specified as the first mediator and attitudes toward aging as the second, with gender, age, and education level included as covariates. In the reverse-order model, the indirect effect through basic psychological needs satisfaction alone was significant (Ind1 = −0.140, *SE* = 0.029, 95% *CI* [−0.201, −0.087]), whereas the indirect effect through attitudes toward aging alone was not (Ind2 = −0.008, *SE* = 0.010, 95% *CI* [−0.031, 0.011]). The sequential indirect effect was also not significant (Ind3 = −0.005, *SE* = 0.006, 95% *CI* [−0.019, 0.007]). In the initially specified model, the sequential indirect effect through attitudes toward aging and basic psychological needs satisfaction was significant. Detailed results are presented in Table 4. Importantly, because the decomposition of indirect associations depends on the specified ordering of mediators, the present results should be interpreted descriptively. A difference in statistical significance between sequential indirect effects across the two models does not demonstrate a significant difference between the pathways and cannot establish the preferable temporal ordering. The cross-sectional data cannot determine which mediator precedes the other; longitudinal or experimental studies are needed to confirm the causal and temporal ordering of the mediators.

## 4. Discussion

The present study examined the association between social media use and psychological symptoms among older adults who reported using at least one social media platform, and tested the indirect roles of attitudes toward aging and basic psychological need satisfaction. Correlation analyses showed that social media use was negatively associated with psychological symptoms, whereas the direct association became non-significant after the mediators were included in the model. Bootstrap analyses indicated that basic psychological need satisfaction significantly mediated this association, and that attitudes toward aging and basic psychological need satisfaction formed a significant sequential indirect pathway. By contrast, attitudes toward aging alone did not significantly mediate the association. Within the specified mediation model, the association between social media use and psychological symptoms showed a significant model-based indirect association via need-related psychological factors, whereas the direct association was not statistically significant. This pattern is correlational and does not establish that the proposed psychological mechanism actually operates or that the variables are causally or temporally ordered. Importantly, because non-users were excluded, the findings reflect variation in the frequency of social media use among existing users, rather than differences between users and non-users. They should therefore not be generalized to the broader older adult population or interpreted as evidence that initiating social media use would reduce psychological symptoms among non-users.

### 4.1. Associations Between Social Media Use and Psychological Symptoms in Older Adults

As people age, they encounter various physical and psychological challenges. This study found a significant link between older adults’ social media use and their psychological symptoms, aligning with previous research (Koh et al., 2024; Naslund et al., 2020). Among existing social media users, older adults who use social media more frequently may have more opportunities to maintain social contact, obtain information, and participate in online interactions, which may be associated with fewer psychological symptoms. Research has shown that older adults who use social media more frequently tend to experience better physical and mental health. This suggests that social media use may be positively associated with better psychological functioning among older adults (Nakagomi et al., 2022; Early & Hernandez, 2021). Additionally, one study analyzing data from the China General Social Survey found that the link between social media use and mental health among older adults was significantly stronger than the relationship with physical health (Fu & Xie, 2021).

It is important to note that the direct link between social media use and psychological symptoms became insignificant once attitudes toward aging and basic psychological need satisfaction were included in the model. This finding indicates that the relationship between social media use and psychological symptoms may be mostly indirect. In other words, using social media more frequently might not directly lead to fewer psychological symptoms. Instead, the psychological significance and need-satisfying aspects of social media use could be more crucial.

### 4.2. Indirect Effect of Basic Psychological Needs Satisfaction

Self-determination theory (SDT) posits that the basic psychological needs satisfaction for autonomy, competence, and relatedness is important for one’s psychological well-being. Based on SDT, the present study explored whether basic psychological need satisfaction may help explain the association between older adults’ social media use and psychological symptoms. As individuals age, they often experience declines in physical function and cognitive abilities. Some older adults may experience difficulties using social media platforms, which may limit their opportunities to maintain contact with family members and friends. Such difficulties may be associated with lower relatedness satisfaction and greater feelings of loneliness. On the other hand, older adults who engage more frequently in social media activities may have more opportunities to choose when and how to communicate with others, which is associated with their basic psychological needs satisfaction (Burns-Lynch et al., 2016). Additionally, social media can help older adults expand their social networks beyond their immediate surroundings, fulfilling their need for social interaction.

### 4.3. Chain Mediation Pathway of Attitudes Toward Aging and Basic Psychological Needs Satisfaction

The results showed that social media use was positively associated with attitudes toward aging among older adults. This association is consistent with previous research. For instance, a survey involving 10,858 older adults indicated that Internet use was significantly linked to attitudes toward aging (X. Wang, 2025). Attitudes toward aging were also negatively correlated with psychological symptoms. However, it is important to note that our measure assessed only the frequency of social and information-oriented behaviors and did not capture exposure to age-related content, age stereotypes, or positive representations of aging. Therefore, although Stereotype Embodiment Theory offers a plausible framework for interpreting this association, the specific stereotype-related mechanism was not directly tested in the present study. Reverse selection is also possible: older adults with more positive attitudes toward aging may be more willing to engage with social media. These possibilities should be examined in future longitudinal or experimental research.

Notably, attitudes toward aging alone did not significantly mediate the association between social media use and psychological symptoms. H2 was not supported, as the indirect pathway through attitudes toward aging alone did not reach statistical significance, with the confidence interval spanning zero. Rather, the results suggest that attitudes toward aging operate primarily in conjunction with basic psychological needs satisfaction as part of a sequential association, rather than as an independent mediator (Thorpe et al., 2014). One possible explanation is that attitudes toward aging represent a relatively broad self-perception, whereas basic psychological need satisfaction is a more proximal psychological resource directly related to emotional adjustment. Thus, positive aging attitudes may be associated with fewer psychological symptoms, mainly when they are accompanied by greater need satisfaction (Pedroso-Chaparro et al., 2023). This null finding could reflect either a genuine absence of the association in the population or a lack of statistical power to detect a small but meaningful effect, given the current sample size. Future research with larger samples is needed to replicate this finding and to clarify whether the association is indeed negligible or simply went undetected.

Further analysis indicated that attitudes toward aging and basic psychological needs satisfaction acted as chain mediators in the association between social media use among older adults and their psychological symptoms. Engaging with social media was associated with more positive attitudes toward aging, which in turn were associated with higher basic psychological need satisfaction (Wurm et al., 2013). One possible interpretation is that older adults with more positive aging attitudes may be more inclined to maintain autonomy, competence, and social relatedness in later life (Steverink et al., 2001). While previous studies have primarily focused on the mediating effects of social environmental factors like social connections and social capital in the relationship between social media use and psychological symptoms in older adults, this study shifts the focus. By examining individual factors such as attitudes toward aging and basic psychological needs satisfaction, we gain a deeper understanding of how older adults’ social media use is associated with their psychological symptoms.

Although these findings may help generate hypotheses for future intervention research, they should not be interpreted as evidence that increasing social media use would reduce psychological symptoms. Because the present study was cross-sectional, included only existing social media users, and did not assess passive use, problematic use, content type, or duration of use, no direct intervention recommendations can be drawn. Future longitudinal or experimental studies are needed to test whether promoting socially engaged and need-supportive forms of social media use can improve psychological outcomes among older adults.

### 4.4. Potential Adverse Effects and Boundary Conditions

It is important to emphasize that the present study assessed only the frequency of social and information-oriented behaviors, using the total social media use score. It did not measure passive use, compulsive use, or problematic use. The beneficial indirect pathways observed here should therefore be understood as conditional, and the following considerations are best framed as directions for future research rather than as conclusions derived from the current data.

The implications of social media use for older adults are not universally positive. The identified pathway—linking social media use to more positive aging attitudes, greater need fulfillment, and fewer psychological symptoms—may only hold under conditions of instrumental, socially engaged use. In contrast, passive or compulsive use can weaken or reverse these benefits. Prior research suggests that excessive digital device reliance relates to negative psychological and physical outcomes (H. J. Lai et al., 2025), and older adults facing a digital divide may experience increased depressive symptoms (Zhu, 2023). Additionally, passive consumption, such as scrolling through feeds or watching short videos, is common among Chinese older adults and may relate to anxiety and cognitive fatigue due to upward social comparisons (J. Huang et al., 2025).

Future research should adopt a balanced perspective to clearly delineate the boundary conditions of beneficial relationships. By integrating multiple methodological approaches, including behavioral tracking data and self-reported measures, subsequent studies should examine the dose–response relationships among social media usage duration, motivational orientation, and psychological symptoms. These efforts should be viewed as hypothesis-generating rather than as a basis for recommending changes in social media use. Only after longitudinal or experimental evidence establishes causality and identifies boundary conditions should such findings inform digital literacy or psychological intervention programs.

### 4.5. Limitations

Several limitations of this study should be acknowledged. First, the cross-sectional design restricts our ability to draw definitive causal inferences. Although significant mediating effects of attitudes toward aging and basic psychological needs satisfaction were identified, the temporal precedence among variables cannot be established. Future research should employ longitudinal or experimental designs to further clarify the causal pathways. Second, self-reported measures may introduce recall and social desirability biases, and common method bias cannot be ruled out despite Harman’s test. The BPNS subscales showed low AVE, indicating limited convergent validity; although retained for its theoretical structure and widespread use, its psychometric properties in Chinese older adults require further examination. Similarly, the Chinese SCL-10 was adapted from the Chinese SCL-90 rather than an independently validated version and has not been systematically validated in this population. Despite acceptable internal consistency (α = 0.874; CR = 0.853), its low AVE (0.374) and variance explained (30.87%) indicate limited convergent validity and only preliminary support for unidimensionality. Thus, SCL-10 findings should be interpreted cautiously, and a validated Chinese SCL-10 for older adults is needed. Third, the eligibility criterion and sampling strategy limit the interpretation and generalizability of the findings. Participants were required to use at least one social media platform, and non-users were therefore excluded. Consequently, the analyses capture associations among existing older social media users rather than differences between users and non-users or associations across the older adult population as a whole. The sample was also limited in size, demographic scope, and geographic coverage. Recruitment from senior activity centers and public parks may have overrepresented relatively healthy and socially active older adults, further limiting generalizability even within the population of older social media users, particularly to those who are less mobile or homebound. Moreover, because the study was conducted in a Chinese context, its applicability to other cultural settings requires further investigation. Future studies should recruit larger and more diverse samples that include both users and non-users, distinguish between social media adoption and frequency of use among existing users, and assess relevant determinants of eligibility and participation. Fourth, this study focused solely on behavioral frequency (i.e., social and information behaviors) without examining qualitative dimensions such as content type, duration, or problematic use. Consequently, the findings cannot specify which online content (e.g., health information, intergenerational communication) is particularly beneficial or harmful for older adults’ psychological symptoms. In addition, we relied on the total score of basic psychological need satisfaction rather than examining autonomy, competence, and relatedness separately. Future research is encouraged to address both limitations by incorporating richer qualitative indicators of social media use and exploring whether the three need dimensions play distinct roles in the proposed mediation model. Lastly, several potentially relevant covariates were not controlled for in the present study, including marital status, health status, living arrangement, socioeconomic status, baseline digital literacy, frequency of offline social contact, as well as the administration mode (online, self-administered offline, and researcher-read questionnaires). These factors may confound the observed associations between social media use and psychological symptoms, and their omission could bias the estimates. Future research should incorporate these variables as covariates or explore their moderating roles to more rigorously isolate the unique contribution of social media use to older adults’ psychological symptoms.

## 5. Conclusions

This study suggests that greater social media use is associated with fewer psychological symptoms among older adults who use social media. The results are consistent with a model in which basic psychological needs satisfaction may serve as a mediator in this relationship. Furthermore, the findings point toward a potential chain mediation pathway, whereby social media engagement is linked to fewer psychological symptoms through sequential associations with attitudes toward aging and basic psychological needs satisfaction. It is important to note that these findings are based on cross-sectional data and cannot establish directionality or causality. Nevertheless, they provide an empirical basis for future longitudinal or experimental research aimed at further exploring these mechanisms and their implications for promoting healthy aging in digital contexts.

## Figures and Tables

**Figure 1:**
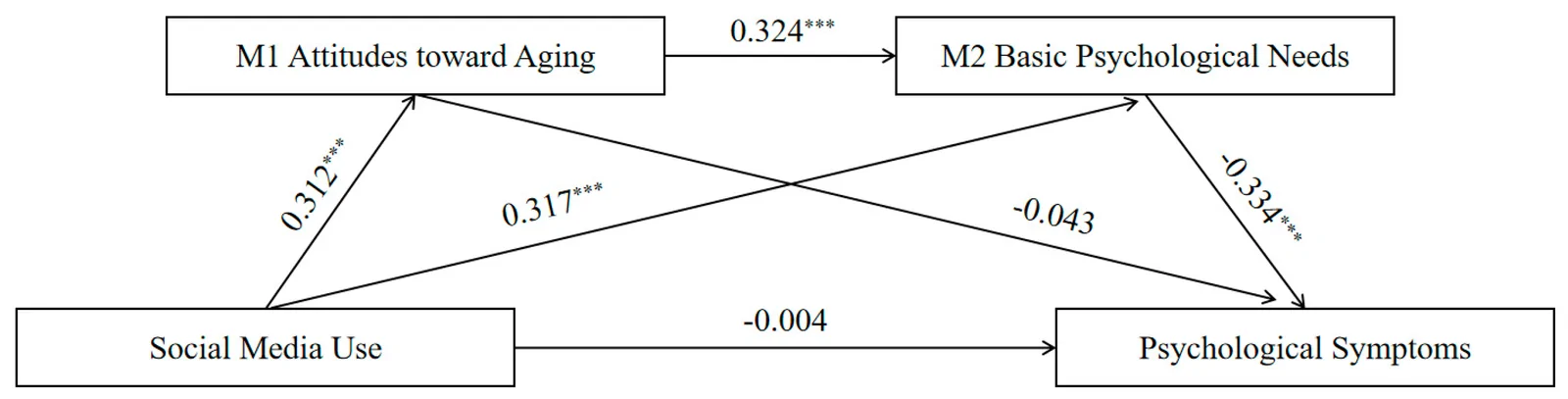
The chain mediation model linking social media use to psychological symptoms through attitudes toward aging and basic psychological needs satisfaction. *** *p* < 0.001.

**Table 1:** Descriptive statistics and correlations among the variables.

	*M*	*SD*	1	2	3	4	5	6
1 Gender	1.64	0.481						
2 Age	67.98	4.996	−0.099					
3 Education level	3.650	0.993	−0.041	−0.052				
4 Social Media Use	68.451	20.595	0.153 **	−0.035	0.306 ***			
5 Attitudes toward Aging	69.549	12.617	0.084	−0.088	0.035	0.316 ***		
6 Basic Psychological Need Satisfaction	96.863	12.263	0.124 *	0.026	0.151 **	0.423 ***	0.392 ***	
7 Psychological Symptoms	13.869	3.630	−0.006	0.006	−0.123 *	−0.201 ***	−0.207 ***	−0.427 ***

Note: Pearson correlations, two-tailed. n = 325. *M* = Mean; *SD* = Standard Deviation. * *p* < 0.05, ** *p* < 0.01, *** *p* < 0.001.

**Table 2:** The regression results of variable relationships.

Predictor Variables	Model 1 (Attitudes Toward Aging)		Model 2 (Basic Psychological Needs Satisfaction)		Model 3 (Psychological Symptoms)	
Social Media Use	β	SE	β	SE	β	SE
	0.312 ***	0.053	0.317 ***	0.056	−0.004	0.051
Attitudes toward Aging			0.324 ***	0.056	−0.043	0.051
Basic Psychological Need Satisfaction					−0.334 ***	0.048
Gender	0.020	0.048	0.062	0.049	0.039	0.042
Age	−0.075	0.051	0.076	0.051	0.012	0.044
Education level	−0.063	0.050	0.053	0.050	−0.046	0.044
F	10.071 ***		23.015 ***		12.588 ***	
R^2^	0.111		0.263		0.191	

Note: All regression coefficients are standardized. Control variables included gender, age, and education level. *SE* = standard error. *** *p* < 0.001.

**Table 3:** Summary of the mediating effects.

	Effect	*SE*	95% *CI*
Total Effect	−0.157	0.049	[−0.254, −0.059]
Direct Effect	−0.004	0.051	[−0.103, 0.096]
Indirect Effect	−0.153	0.032	[−0.219, −0.095]
Ind1	−0.013	0.016	[−0.047, 0.017]
Ind2	−0.106	0.026	[−0.161, −0.060]
Ind3	−0.034	0.011	[−0.057, −0.017]

Note: *SE* = standard error; *CI* = confidence interval. Ind1: Social Media Use → Attitudes toward Aging → Psychological Symptoms; Ind2: Social Media Use → Basic Psychological Needs Satisfaction → Psychological Symptoms; Ind3: Social Media Use → Attitudes toward Aging → Basic Psychological Needs Satisfaction → Psychological Symptoms.

**Table 4:** Summary of mediating effects in the alternative reverse-order mediator model.

	Effect	*SE*	95% *CI*
Total Effect	−0.157	0.049	[−0.254, −0.059]
Direct Effect	−0.004	0.051	[−0.103, 0.096]
Indirect Effect	−0.153	0.032	[−0.219, −0.095]
Ind1	−0.140	0.029	[−0.201, −0.087]
Ind2	−0.008	0.010	[−0.031, 0.011]
Ind3	−0.005	0.006	[−0.019, 0.007]

Note: SE = standard error; CI = confidence interval. Ind1: Social Media Use → Basic Psychological Needs → Psychological Symptoms; Ind2: Social Media Use → Attitudes toward Aging → Psychological Symptoms; Ind3: Social Media Use → Basic Psychological Needs → Attitudes toward Aging → Psychological Symptoms. Indirect effects were estimated with 5000 bootstrap samples.

## Data Availability

The datasets used and analyzed during the current study are available from the corresponding author on reasonable request.

## Abbreviations

The following abbreviations are used in this manuscript:

BPNS	Basic Psychological Needs Scale
CI	Confidence interval
M	Mean
SCL-10	Symptom Checklist-10
SCL-90	Symptom Checklist-90
SD	Standard Deviation
SE	Standard Error
SDT	Self-determination theory
